# A Sequential Three-Phase Pathway Constitutes Tracheary Element Connection in the *Arabidopsis*/*Nicotiana* Interfamilial Grafts

**DOI:** 10.3389/fpls.2021.664342

**Published:** 2021-07-05

**Authors:** Zhuying Deng, Huiyan Wu, Tianlin Jin, Tingting Cai, Mengting Jiang, Mi Wang, Dacheng Liang

**Affiliations:** ^1^Hubei Collaborative Innovation Center for Grain Industry, School of Agriculture, Yangtze University, Jingzhou, China; ^2^Engineering Research Center of Ecology and Agricultural Use of Wetland, Ministry of Education, Yangtze University, Jingzhou, China

**Keywords:** heterograft, TE segmentation, short TEs, cellular deposits, aligning, spiraling tracheary elements, compatibility, incompatibility

## Abstract

Scion-rootstock union formation is a critical step toward the functional assemblage of heterogeneous plants. Interfamilial scion-rootstock interaction often results in graft incompatibility during the assemblage process, and the underlying mechanisms are largely unknown. In this study, we reported that tracheary element (TE) remodeling, including TE segmentation and deformation, rather than *de novo* formation from callus or adjacent tissues, took place at the early stage of grafting interface between *Arabidopsis thaliana* and *Nicotiana benthamiana* (*At*/*Nb*). Following cellular deposits, the short TEs from both partners were overlapping, dependent on the homogeneity of contacting TEs, with each other. Without overlapping, the TEs at the interface would grow laterally, and the TEs above and below the interface would undergo self-fusion to form insulating spiraling bundles. Finally, the overlapping TEs constituted a continuous network through alignment. Our results provide a definitive framework for the critical process of TE behavior in the *At*/*Nb* distant grafts, including (1) segmentation and/or deformation, (2) matching, overlapping, and cellular deposits, and (3) aligning or spiraling. These insights might guide us in the future into constructing more compatible distant grafts from the perspective of TE homogeneity.

## Introduction

Grafting is an ancient technique that allows an artificial combination of two different parts of the plants (rootstock and scion) into a living symbiont and has been widely applied in horticultural and biological research. For example, it was employed to study the physiological mechanisms of shoot branching (Turnbull et al., 2002), flowering (Corbesier et al., 2007; Lin et al., 2007; Yoo et al., 2013; Zhu et al., 2016), vegetable soil-borne disease (Lee et al., 2010; Schwarz et al., 2010; Vitale et al., 2014), nutrient allocation (Rus et al., 2006; Lin et al., 2008; Pant et al., 2008; Kumar et al., 2017; Li et al., 2017; Ohkubo et al., 2017), and other physical processes.

Construction of a successful graft with various grafting approaches enables the studies of interorgan or inter-tissue communication. For example, cotyledon grafting showed that a wild-type (WT) cotyledon, as a source of florigen, can rescue the late flowering phenotype of the *ft-10* mutant by supplying the mobile FLOWERING LOCUS T (FT) protein (Yoo et al., 2013). Inflorescence grafting was employed to study information flow between the WT and the *acl1-1* mutant with the premature arrest of the inflorescence meristem (Tsukaya et al., 1993). Recently, stem grafting was also used to demonstrate the movement of the antiflorigen *NsCET1* mRNA from tobacco to tomato (Huang et al., 2018). Another study involving the tobacco and tomato heterograft has adopted the same method to identify transcripts that move from scion (*Nicotiana*) to rootstock (tomato) (Xia et al., 2018). Grafting in the hypocotyl tissue is a good way to demonstrate root-to-shoot signaling, e.g., long-distance mobile silencing (Brosnan et al., 2007; Liang et al., 2012), root-to-shoot signals for branching (Turnbull et al., 2002), and root-derived *bps* signaling for regulating shoot development (Van Norman et al., 2004). Although the grafting technique is widely applied for different purposes, the critical principle underlying grafting compatibility is poorly understood.

Usually, grafting partners need to be taxonomically (near-)equivalent. This rule dictates that most grafts are made within the same species (intraspecific) or the same genus (interspecific). With the increase of taxonomic distance, grafts are becoming less possible. As such, the intrafamilial grafts are found to be rarely compatible, and interfamilial grafts are considered always incompatible (Goldschmidt, 2014). However, some existing and emerging studies have shown the grafting samples that violate the above general rule. For example, an early study attempted to construct interfamilial grafts between *Helianthus tuberosus* (Asteraceae family) and *Viciafaba* (Fabaceae family) and found the cytoplasmic components of the connected cells at the graft union showed no signs of degeneration, suggesting a certain degree of compatibility/incompatibility (Kollmann and Glockmann, 1985). Another study made an interfamilial graft between *Arabidopsis* (Brassicaceae family) and tomato (Solanaceae family) in which the *Arabidopsis* scion was able to flower and develop seeds (Flaishman et al., 2008). Furthermore, interfamilial grafts seem to be able to perform the physiological function; for instance, interfamilial graft between periwinkle (Apocynaceae family) and apple (Rosaceae family) was able to transmit a phytoplasma pathogen from apple to periwinkle (Aldaghi et al., 2007). A more recent grafting advancement has showed that more than 70 species from 38 families can form an interfamilial combination with a Solanaceae family member, *Nicotiana* (Notaguchi et al., 2020). Therefore, these studies suggested that distant graft, including interfamilial graft, could be feasible, although many of them showed a certain degree of incompatibility/compatibility (Kollmann and Glockmann, 1985; Flaishman et al., 2008).

One of the fundamental questions is how the grafting partners are joined at the grafting interface. Kollmann and Glockmann (1985) showed that the contacted cells in the union of *Helianthus*/*Vicia* interfamilial graft were connected by simple and branched plasmodesmata, suggesting the two species communicated at the supracellular level. Presumably, a similar situation could also occur at the parenchyma cells in the graft union of *Arabidopsis*/tomato combination. Probably, plasmodesmata could complement the intercellular transport when there is a lack of vascular connection between the two (Flaishman et al., 2008). Alternatively, cell wall remodeling mediated by β-1,4-glucanase can facilitate cell–cell adhesion (Notaguchi et al., 2020).

Formation of the vascular connection, as laid in the majority of grafting research, is the principal step in establishing the compatibility of graft combinations (Yeoman, 1984; Pina and Errea, 2005). The proliferation of callus during graft union formation, together with the position of newly formed xylem, led to the widespread hypothesis that vascular connections are achieved through the production of the secondary xylem and phloem from the new vascular cambium differentiated from callus (Moore, 1983; Pina and Errea, 2005; Baron et al., 2019). However, some exceptions to this hypothesis also exist in the literature; for instance, there is no vascular redifferentiation from callus cells in the interfamilial graft between *Sedum telephoides* (Crassulaceae) and *Solanumpennellii* (Solanaceae) (Moore and Walker, 1981), implying that xylem connection may be achieved through another cellular process. In addition, an early study by Copes (1980) found that irregular tracheid arrangement in the pine tree grafts was associated with grafting incompatibility, suggesting the tracheid/TE behavior plays a very important role in scion–rootstock interaction.

Grafting compatibility, according to the restricted definition (Yeoman et al., 1978; Hartmann et al., 2011), refers to the union of the vascular elements of stock and scion; conversely, incompatibility refers to vascular discontinuity at the graft union. In this study, we found there were three groups of *At*/*Nb* interfamilial grafts that could be classified into compatible and incompatible grafts based on their vascular connection. To understand how compatibility/incompatibility arose from the same heterografting combination, we focused on examining the behavior of TEs at the grafting union in both compatible and incompatible grafts. Our new approach revealed that TE segmentation and deformation rather than redifferentiation at the grafting interface constituted the first key step toward establishing the grafting union. The matched TEs deposited with the membrane-like substances could overlap and align to form a compatible graft, whereas the non-homogenous TEs repelled each other at the interface and grew into highly spiraled TE bundles that heralded graft incompatibility.

## Materials and Methods

### Plant Material and Growth Conditions

For each batch of grafting, around 300 seeds of the wild-type *Arabidopsis thaliana* (*At*, Col-0), the *35S-GFP* transgenic line (Brosnan et al., 2007), and the wild-type *Nicotiana benthamiana* (*Nb*) were surface-sterilized in chlorine gas for 1 h and then plated on the sterile Murashige and Skoog (MS) medium, supplemented with 3% (w/v) sucrose. Plates were grown longitudinally in a growth room under long-day conditions (16-h light/8-h dark) set at 22–23°C.

### Micrografting

**Figure 1:**
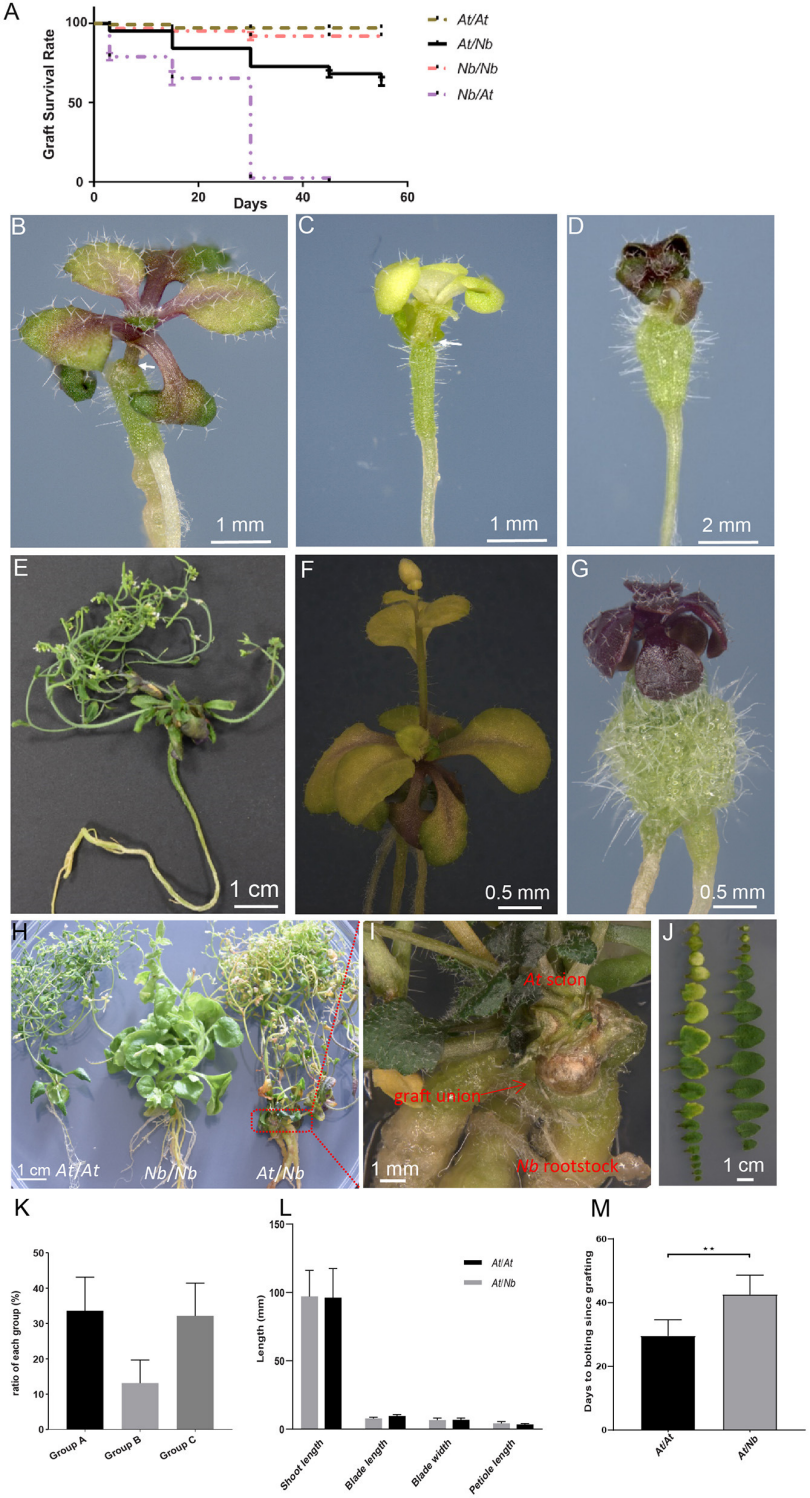
Three types of *At*/*Nb* heterografts grown under aseptic conditions. **(A)** The survival rate of self-grafts and heterografts. **(B)** A representative plant of group A grafts that show mild stress at 30 DAG. **(C)** A representative plant of group B grafts with a chlorotic phenotype at 30 DAG. **(D)** A representative plant of group C grafts, showing highly retarded growth or quiescent state at 30 DAG. **(E)** A 135 DAG graft from group A. **(F)** A 45 DAG graft from group B. **(G)** A 45 DAG graft from group C. **(H)** Grafts at 155 DAG. Left, the *At* self-graft; middle: *Nb* self-graft; right, *At*/*Nb* graft. **(I)** The graft union of the *At*/*Nb* graft in **(G)**. **(J)** Leaves from *At*/*Nb* (left) and *At* self-graft (right). **(K)** The ratio of three groups at 30 DAG. Data were from six independent experiments, and the number of grafts is shown in Table 1. **(L)** Shoot, root, and leaf measurements in *At* self-grafts and *At*/*Nb* heterografts (group A). Data were shown as the mean ± SD (*n* = 5 for each grafting type). **(M)** Flowering-time comparison between self-grafts and group A grafts (*p* < 0.01). Data were shown as the mean ± SD (*n* = 8 for each grafting type). An arrow indicated the graft junction. **p < 0.01.

The grafting procedure was described in detail by Andersen et al. (2014). Young seedlings that were grown on the MS medium for 7–9 days were no more than 8 mm in total length for *Nb* plants and 2 cm for *At* plants, and all with long straight hypocotyls were used for grafting (heterografts and self-grafts). The cut was made halfway from the base of hypocotyl on the moisturized Whatman paper. The scion and the rootstock with a smooth-cut surface were pushed together with a certain tension. Grafts were grown on moisturized Whatman paper for 2 days, and then the grafts were gently lifted with forceps and placed vertically onto the MS medium with 1% agar and 3% sucrose (w/v) in the growth room (16-h light/8-h dark) at 22–23°C. Totally, there were more than 20 batches of heterografts and self-grafts that were made. We followed the full growth of 449 *At*/*Nb* grafts from six batches, and the results are presented in Table 1 and Figure 1K.

**Table 1:** Three types of *At*/*Nb* grafts at 30 DAG: mild-stress grafts, chlorotic grafts, and quiescent grafts.

**No. of grafts**	**Mild-stressed**	**Percentage (%)**	**Chlorotic**	**Percentage (%)**	**Quiescent**	**Percentage (%)**
125	37	29.6	15	12	47	37.6
77	17	22.08	16	20.78	27	35.06
80	33	41.25	16	20	20	25
47	21	44.68	6	12.77	8	17.02
58	23	39.66	2	3.44	21	36.21
62	15	24.19	6	9.68	26	41.94

### Symplastic and Apoplastic Dyes for Vascular Reconnection Analysis

Xylem and phloem connectivity was measured with acid fuchsin and 5(6)-carboxyfluoresceindiacetate (CFDA) loading, respectively. About 1% (w/v) acid fuchsin solution (sigma) was introduced into the vascular system of heterografts and self-grafts by submerging the cut end of roots (the cut was made 2–3 mm above the root tip) in the solution at room temperature (Flaishman et al., 2008; Yin et al., 2012). Make sure that the hypocotyl of rootstock was kept away from the solution. The cotyledon of the scion was examined under a bright-field microscope after 30–60-min incubation. Xylem connection can be reflected by the vein stains. For each time point, three experiments with no <15 individual grafts were conducted.

For CFDA staining, a fresh working solution of 5-μM CFDA in distilled water was prepared from a 1-mM stock solution in dimethyl sulfoxide (DMSO). A syringe needle was used to gently puncture the newly grown leaf. The amount of 0.25 μl of 5-μM CFDA was pipetted onto the lightly damaged leaf and kept in the darkness at room temperature. Alternatively, the CFDA solution was applied to the lower part of roots that were put on the parafilm and incubated in the darkness at room temperature for 1–2 h (Jiang et al., 2019). Phloem connection can be reflected by the rootstock fluorescence when CF was loaded in the scion. The scion fluorescence was checked when the dye was loaded into the rootstock. For each time point and CF loading site (scion and rootstock, respectively), three experiments with 11–15 individual grafts were conducted. The fluorescent signal was detected and imaged with a Nikon ECLIPSE Ni fluorescence microscope and a Zeiss Axio Zoom V16 fluorescence microscope with a 1 × /2 × lens.

### Scanning Electron Microscopy and Histological Sectioning

For TE detection, the leaves and the roots of grafts were removed, and the hypocotyl region, including the upper and lower regions of graft union, was chosen and immediately fixed in 4% paraformaldehyde in a 1X PBS buffer for 30 min. The fixed samples were rinsed three times with PBS and dissected to remove the tissues adjacent to the vascular bundles under a dissecting microscope. The longitudinally dissected vascular samples were further washed in 1.2% Triton X-100 for 15 min, and then rinsed in PBS for 15 min. The washing step can be repeated additional times if necessary. The dissected materials were dehydrated for 15 min each in an ethanol series of 25, 50, 75, and 100% ethanol. After three times of washes with absolute ethanol, the samples were dried in a −20°C, low-vacuum drier (CHRIST). The dried samples were then mounted on stubs with pre-mounted carbon conductive films and coated with gold. Examination of the samples was performed with a MIRA3 field-emission scanning electron microscope from TESCAN.

For histological analysis, the hypocotyl region, including upper and lower regions of graft union, was collected from 100 DAG (day after grafting) plants and fixed with 4% paraformaldehyde in a 1X PBS buffer overnight at 4°C after vacuum infiltration. The samples were dehydrated and embedded in Paraplast, following a standard method. The samples were sectioned longitudinally into 10-μm-thick sections (RM2235, Leica) and mounted on glass slides. For staining, the sections were dewaxed and incubated in 0.5% Toluidine blue O solution for 5 min, in 0.5% congo red solution for 30 min, or in 1% Safranin O solution for 2 h. The stained samples were rinsed with water and then briefly rinsed once with 95% ethanol. Then, the sections were imaged with a Nikon ECLIPSE Ni microscope.

### Confocal and Epi-Fluorescence Microscopy

Fresh hypocotyls were cut vertically in half with a razor blade for the grafts <20 DAG. For grafts over 20 DAG, the hypocotyl was cut vertically in three parts. These sections were imaged with a Leica SP8 confocal laser-scanning microscope (Leica Microsystems), equipped with a 40 × water immersion objective. Images were acquired with the Leica LAS X software. For epi-fluorescence in the whole plants, the image was taken by a Zeiss stereoscopic microscope (Axio Zoom V16) (Jiang et al., 2019).

## Results

### Development of *A. thaliana* (*At*)/*N. benthamiana* (*Nb*) Heterografting System

*Arabidopsis thaliana* (*At*) and *Nicotiana benthamiana* (*Nb*), belonging to the rosids and asterids, respectively, have distinct genomic information; thus, grafts between the two most likely displayed incompatibility. In our initial attempt to construct an *At-Nb* graft by micrografting (Andersen et al., 2014), we were able to generate an *At*/*Nb* heterograft with *At* as the scion and *Nb* as the rootstock (Supplementary Figure 1). However, a reciprocal graft with *Nb* as scion and *At* as rootstock proved to be very challenging, with a scant grafting survival rate of <1% compared with *At* or *Nb* self-grafts (Figure 1A, Log-rank test, *p* < 0.0001). Although the *At*/*Nb* grafts showed a lower grafting survival rate than that of the self-grafted *At* and *Nb* (Supplementary Figure 1), this combination still resulted in a more than 60% grafting survival rate within 60 days (Figure 1A). The low success rate of the *At*/*Nb* combination partly resulted from adventitious root formation from the *At* scion. Adventitious roots first emerged from a hypocotyl tissue at about 7–10 days after grafting (DAG). The emerging roots were immediately excised, but this removal did not stop their regeneration, and up to 29% of the scions with an average of 23% developed adventitious roots at 24 DAG (Supplementary Table 1). Since adventitious roots can bypass the *At*/*Nb* graft union and fully support the growth of the *At* scion, grafts with any signs of adventitious roots were discarded.

Phenotypically, the remaining grafts could be classified into three distinct groups: the mild-stressed (Figure 1B, Group A), the chlorotic (Figure 1C, Group B) and the retarded grafts (Figure 1D, Group C). About 33% of the grafts (22–45% in different graft batches) showed mild-stressed phenotypes (Table 1, Figure 1K). Under the aseptic condition, the mild-stressed grafts could recover to normal growth and set seeds (Figures 1E–I). Around 80% of the chlorotic grafts also gradually turned green, flowered, and set seeds (Figure 1F). However, the retarded grafts, accounting for 32% of the total (Table 1, Figure 1K), remained in “quiescence” and eventually died (Figure 1G).

The phenotypes of the mild-stressed scions were essentially similar to that of the *At* self-grafts in respect to the leaf parameters (Figures 1J,L). The *At*/*Nb* scion generated slightly more leaves than the *At* self-grafts (Figures 1H,J), which could be due to the late flowering of the *At*/*Nb* grafts (Figure 1M). And, most obviously, it took 140 ± 20 days for the Group A grafts to complete their entire cycle, far longer than that of the *At* self-grafts (60 ± 10 DAG).

### Detection of Vascular Reconnection in *At*/*Nb* Grafts by Symplastic and Apoplatic Dyes

**Figure 2:**
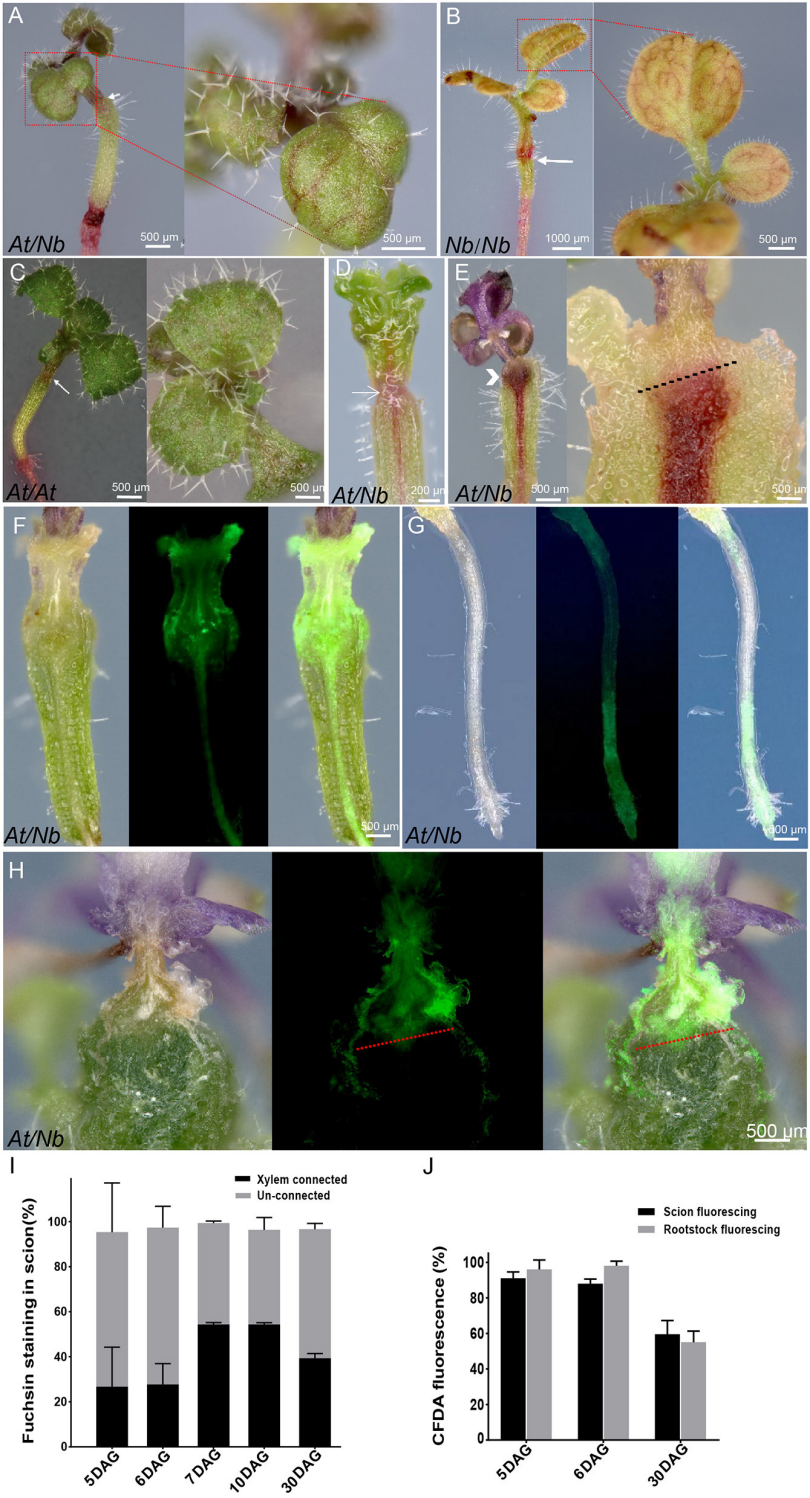
Apoplastic and symplastic dye loading in *At*/*Nb* grafts/ (A–E) 1% acid fuchsin loading. **(A)** Fuchsin staining in the veins of 10-DAG group A plants. **(B)** 7 DAG of *Nb* self-graft. **(C)** 7 DAG of *At* self-graft. **(D)** 5-DAG *At*/*Nb* plants (group A). **(E)** 30 DAG of a quiescent graft (group C). Chevron indicated the accumulated fuchsin in the graft union of retarded grafts. An arrow indicated the graft union. The black dotted line indicated where the fuchsin stops. **(F–H)** CFDA loading in the *At*/*Nb* grafts. **(F)** CFDA loading in a 5 DAG graft (groups A and B). Left: the longitudinal section under the bright field. Middle: the same section under the fluorescence. Right: the overlay image of left and middle images. **(G)** The CFDA signal in the root of 5 DAG *At*/*Nb* graft (groups A and B). Left: the root under the bright field. Middle: the same root under the fluorescence. Right: the overlay image of the left and the middle. **(H)** CFDA loading in a quiescent graft (30 DAG, group C). Left: the longitudinal section under the bright field. Middle: the same section under the fluorescence. Right: the overlay image of the left and the middle. The red dotted line indicated where the CF stops. **(I)** Fuchsin-staining efficiency in the scion when loaded in the rootstock. Data were shown as the mean ± SD (*n* = 7–15 grafts for each time point). **(J)** CF fluorescing efficiency in the scion (loaded in the rootstock) and rootstock (loaded in the scion). Data were shown as the mean ± SD (*n* = 7–15 grafts for each time point per loading type).

Once the grafting between *At* and *Nb* became feasible, we analyzed the vascular connection between *At* scion and *Nb* rootstock. We first used the apoplastis dye, acid fuchsin, to investigate whether the xylem was connected or not (Sano et al., 2005; Yin et al., 2012). By 30–60 min after application of fuchsin to the root tips, the red stain was readily seen in the veins of cotyledons of 7- and 10-DAG (Figure 2A), and the staining intensity was comparable to that of *Nb* self-grafts (Figure 2B). The stain was even stronger than that of *At* self-grafts (Figure 2C), implying a stronger rootstock in the *At*/*Nb* grafting combination. Although the stain was not obvious in the veins of 5-DAG plants, longitudinal sectioning showed that some red dye had moved through the graft union (Figure 2D), but the ratio of scion staining was much higher at 7 DAG (Figure 2I), suggesting that effective xylem reconnections occurred within 7 DAG. Since the xylem connection for water and water-dissolved nutrient transport between scion and rootstock is fundamental to graft survival, the same method was used to examine those grafts that displayed a “quiescent” state (Group C, Figure 1D) in which the scion nearly ceased growth, but rootstock is still alive at 30 DAG (Figure 1G). These retarded grafts showed no visible stains in the leaves, even after prolonged incubation of up to 3 h, indicating no xylem reconnection. Further longitudinal sectioning through the grafts showed fuchsin accumulations below the graft junction (Figure 2E), further suggesting that graft failure in the *At*/*Nb* combination could be attributed, at least partially, to the lack of xylem reconnection between scion and rootstock.

Phloem is an essential system for transporting not only photo-assimilates but also proteins, RNAs, and other signaling molecules. Thus, we sought to detect phloem connectivity, using the phloem-mobile fluorescent tracer carboxyfluorescein (CF). After applying the non-cleaved form, 5(6)-carboxyfluoresceindiacetate (CFDA) to the leaves of 5 DAG grafts, the CF signal could be seen in hypocotyls and roots (Figures 2F,G), and the majority of grafts showed such fluorescence at this time (Figure 2J). These results suggest that phloem reconnection occurred around 5 DAG, similar to *Arabidopsis* self-grafts (Melnyk et al., 2015). However, by 30 DAG, the overall fluorescing efficiency was reduced (Figure 2J), and this reduction was mainly attributed to the quiescent grafts (Group C in Figure 1) in which the up- and down-streaming of CF were blocked at the graft union (Figure 2H). Only for a small part of these grafts (2 out of 11), the connection looked weak (Supplementary Figure 2). These results suggested that phloem connection is essential, but not sufficient for active growth of *At*/*Nb* graft.

### Local TE Segmentation and Cellular Deposits at the Grafting Union

**Figure 3:**
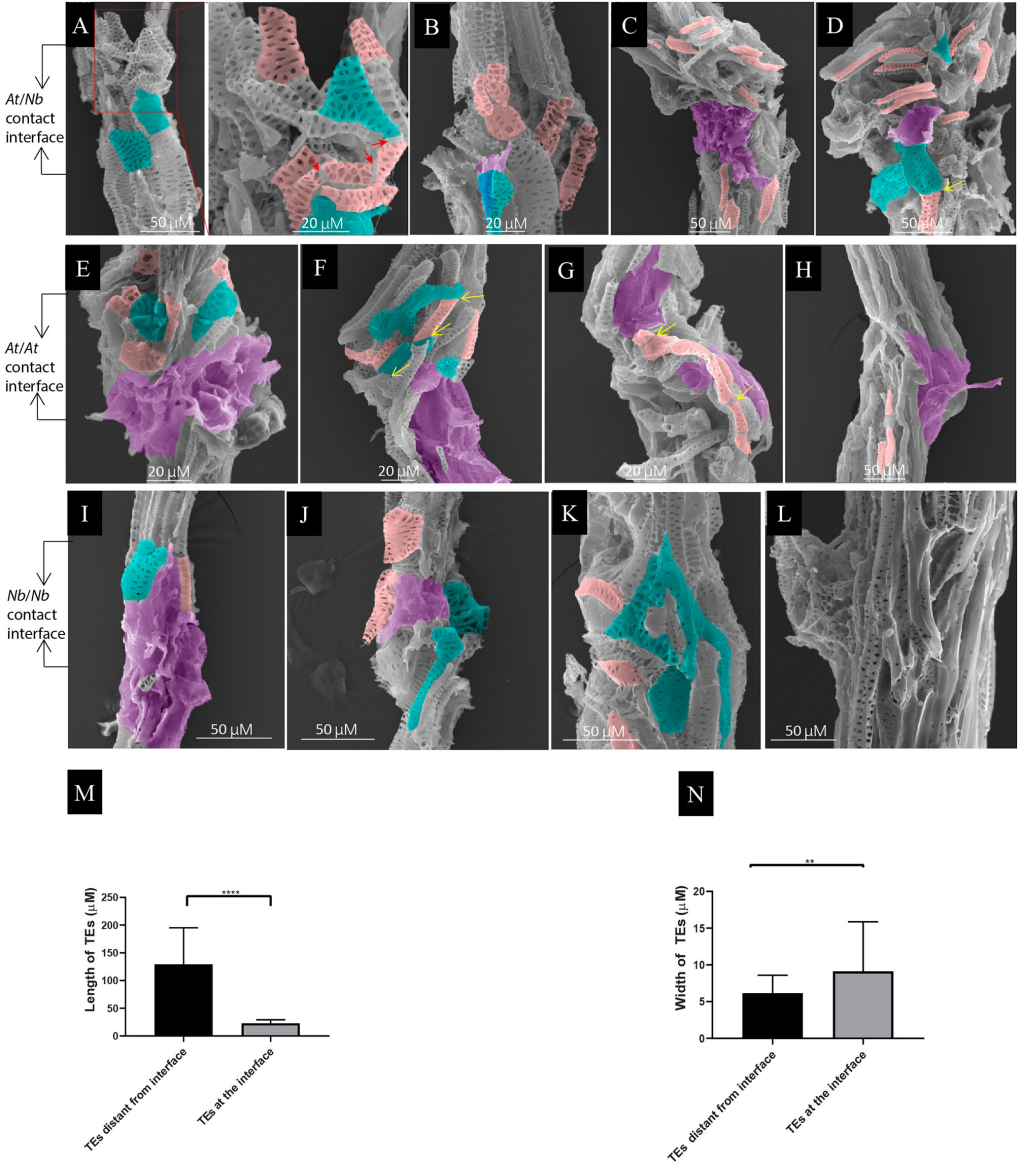
Local TE segmentation and cellular deposits on the TEs during graft union formation. **(A)** *At*/*Nb* graft union at 7 DAG. The shape of some segmented TEs became irregular. Noted that the TEs from *At* became widened, and the end became attenuated. The red arrows indicated the cracks from a single TE. **(B)** *At*/*Nb* graft union at 9 DAG. Noticed that the deposit of membrane-like patches on the TEs occurred at this stage. **(C)** *At*/*Nb* graft union at 14 DAG. More small, short TEs accumulated at the interface, and larger membrane-like patches covered the interface. **(D)** *At*/*Nb* graft union at 21 DAG. Short TEs further accumulated at the interface, and some of them began to fuse indicated by the yellow arrows. **(E)** *At* self-graft union at 7 DAG. Small segmented and deformed TEs accumulated at the interface, and the whole interface was covered by the cellular deposits. **(F)** *At*/*At* graft union at 9 DAG. Noticed the head-to-head fusion and overlapping between TEs indicated by the yellow arrow. **(G)** *At* self-graft union at 14 DAG. Short TEs were accumulated around the union. Noticed that a single longer TE consisting of fused short TEs crossed the interface. **(H)** *At* self-graft union at 21 DAG. TEs were connected and only a few small TEs were left. Partial areas of the interface were covered by the cellular deposits. **(I)** *Nb* self-graft union at 7 DAG. Small segmented and deformed TEs accumulated at the interface, and the whole interface was covered by the cellular deposits. **(J)** *Nb* self-graft union at 9 DAG. Partial areas of the interface were covered by the cellular deposits. **(K)** *Nb* self-graft union at 14 DAG. The short TEs and the irregular TEs shaped into an edge-matching network. **(L)** *Nb* self-graft union at 21 DAG. TEs were well-connected. **(M)** TE length at the interface. Data were shown as the mean ± SD (*n* = 11). Student's *T*-test was used to generate the *p-*value; ****indicated *p* < 0.0001. **(N)** TE width of *At* at the interface of *At*/*Nb* graft. Data were shown as the mean ± SD (*n* = 11). Student's *T*-test was used to generate the *p-*value. **indicated *p* < 0.01. The pink rendering corresponds to the segmented or short TEs. The green-blue rendering corresponds to irregular TEs. The magenta rendering corresponds to the cellular deposits.

The critical role of xylem connection in grafting compatibility drove us to explore the behaviors of tracheary elements during the formation of the graft union. From 7 to 21 DAG, we observed many small, short TEs accumulated at the interface in both *At*/*Nb* heterografts and self-grafts of *At* and *Nb* (Figures 3A–L), and their length was much shorter than the regular length (Figure 3M). Particularly, the mature TEs around the union of 7-DAG *At*/*Nb* graft were undergoing the segmentation process manifested by the presence of the “cracks” in between (Figure 3A). The width of some TEs was slightly increased (Figure 3N), resulting in an irregular shape (Figures 3A,B,E,I–K). At 14 DAG, the short TEs were joined together into longer continuity that crossed the graft union in the *At* self-graft (Figure 3G), and a similar situation also occurred to *Nb* self-grafts in which the smaller and irregular TEs were joined together by forming an edge-matching network (Figure 3K). However, in the *At*/*Nb* grafts, more short TEs from both the *At* and *Nb* accumulated around the graft union, suggesting the production of short TEs is intensifying due to *At*-*Nb* interaction (Figure 3C). Till 21 DAG, part of the short TEs was joined (Figure 3D) while the TEs in *At* and *Nb* self-grafts were usually well-connected (Figures 3H,L).

Another obvious phenomenon of the union development is the deposits of membrane-like substances on the TEs of graft union (Figures 3B–J). At 7 DAG, the *At*/*Nb* graft union did not show the sign of cellular deposits when washed with 1.2% Triton X-100 solution. From 9 DAG, the amount of deposits increased sufficiently such that the entire interface was covered (Figures 3C,D). The same process was happening in both *At* and *Nb* but about 2 days earlier (Figures 3E,I). These results suggested that the interaction between *At* and *Nb* gave rise to two sequential events: TE segmentation and cellular deposits. Finally, when the short TEs successfully formed a network, these deposits then disappeared or declined (Figures 3G,H,K,L), suggesting the potential role of cellular deposits in TE matching and repairing.

### TE Overlapping in Growth-Active Grafts

At 9 DAG of *At* self-grafts, we observed the head-to-head fusion (Figure 3F) or overlapping between adjacent TEs. And till 21 DAG, many long TEs crossed the interface, suggesting a strong reunion between scion and rootstock (Figures 3H,L). In *Nb* self-grafts, there were also existing such two types of connections (Figure 3K). In *At*/*Nb* grafts, the TE connection occurred although weakly as early as 5 DAG indicated by fuchsin-loading experiment (Figure 2D), but we did not detect obvious TE-TE fusion at an early stage of grafting, e.g., at 7 or 9 DAG (Figures 3A,B). We only observed the head-to-head contact at the 7–9 DAG *At*/*Nb* grafts, and these contacts seemed loose and might not be sufficient to constitute a supportive xylem network. We wondered if any alternative form of connection could be responsible for connectivity in *At*/*Nb* grafts.

**Figure 4:**
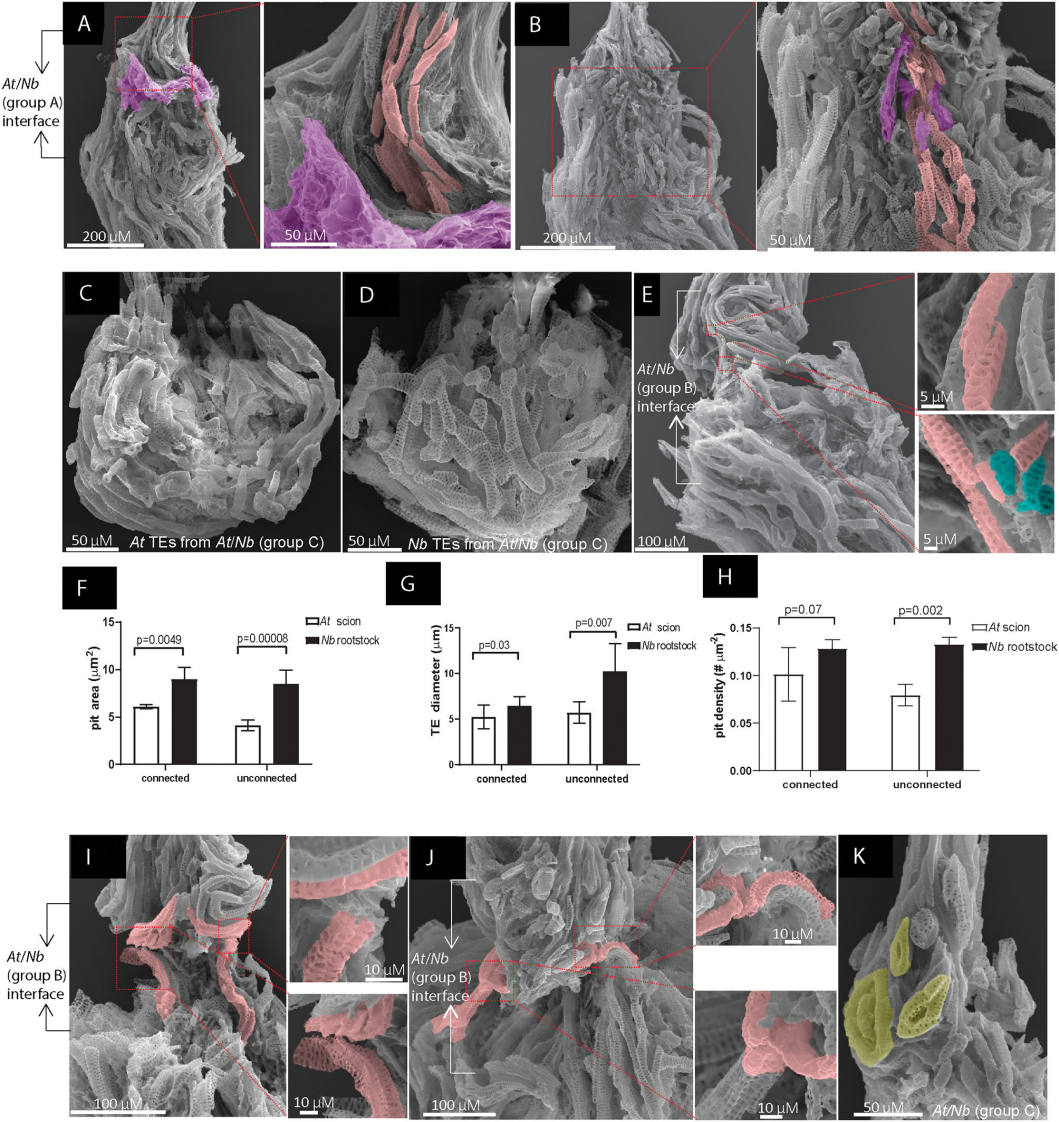
TE overlapping and alignment in *At*/*Nb* grafts. **(A)** TEs at the grafting union of Group A graft washing with 1.2% Triton X-100. Right: the short TEs above the union were overlapping and aligned. **(B)** The same graft in **(A)** was washed with 5% Triton X-100 to reveal the grafting interface. Right: the reticulate TEs of *At* and *Nb* were overlapping and aligned. **(C)** The horizontally growing TEs of *At* at the interface of group C grafts. **(D)** The horizontally growing TEs of *Nb* at the interface of group C grafts. **(E)** The grafting interface in group B grafts. Right top: overlapping of the top end of an *Nb* TE with an *At* TE. Right bottom: overlapping of the bottom end of the same *Nb* TE with other *Nb* TEs. **(F)** Comparison of the pit area in the connected (*n* = 7) and unconnected TEs at the interface (*n* = 9). **(G)** Comparison of the TE diameter in the connected (*n* = 6) and unconnected TEs at the interface (*n* = 6). **(H)** Comparison of the pit density in the connected (*n* = 9) and unconnected TEs at the interface (*n* = 9). **(I)** contact of the non-homogenous TEs. Right: the repelling pattern was enlarged. **(J)** Two patterns formed in the same graft. Right Top: contact of the homogenous TE. Right Bottom: the non-homogenous TEs repelled each other. **(K)** The circled TEs formed above the grafting interface of group C grafts due to self-fusion. The pink rendering corresponds to the short TEs. The green-blue rendering corresponds to irregular TEs. The magenta rendering corresponds to the cellular deposits. The yellow rendering corresponds to the self-fused TEs. Data in **(F–H)** were shown as the mean ± SD. *p-*value was calculated by Student's *T*-test.

The majority of the grafting interface was covered by the membrane-like patches (Figures 3B–J), thus blocking the details of the connection between the two parts. Since the concentration of Triton X-100 was strongly associated with membrane destruction (Koley and Bard, 2010), we further increased the Triton X-100 detergent to 5% to remove the membrane-like patches. As shown in the Figures 4A,B, the membrane-like patches were efficiently removed. The unveiled interface showed us that the short TEs were aligned vertically *via* overlapping to form a continuous network (Figure 4B). Below and above the interface, the homogenous TEs were highly aligned *via* both overlapping and head-to-head connection (Figure 4A). However, for the group C grafts, the short TEs at the contact interface grew horizontally, and no alignment occurred (Figures 4C,D), which is agreeing with the fuchsin-loading experiment. In addition, consistent with the phenotypes of group B grafts, only partial alignment occurred between the *At* scion and *Nb* rootstock (Figure 4E), strongly suggesting the extent of TE alignment was correlated with grafting success.

It is noticeable that the overlapping elements were structurally matchable in terms of a TE diameter, a pit area, and pit density (Figures 4A,B,E). We measured the overlapping TEs between the two species and found the pit diameter, density, and pit area were similar to each other (Figures 4F–H). Although the pit area in the TEs of group A was different (Figure 4F), the substantial difference in which the pit area of the *Nb* TEs was more than 2-fold larger than that of the *At* TEs would render the connection impossible (Supplementary Figure 3A). Similarly, the diameter of TEs from the partners needed to be similar; otherwise, there was no connection even though the TEs were placed in juxtaposition to each other (Supplementary Figure 3B).

Pit patterning was also an important factor in TE overlapping. In *At*/*Nb* grafts, we mainly detected the overlapping TEs with reticulate patterning (Figures 4B,E). When the TEs with distinct pitting patterns approached, they apparently repelled with each other instead of overlapping with each other, resulting in a curved arrangement (Figure 4I). This situation could occur in the same graft with different shapes of TEs; in Figure 4J, the similar TEs formed a straight head-to-head contact; however, the TEs with different pitting patterns curved when close to each other. All these results strongly indicated that the short TEs were engaged in a recognizing process by which the homogenous TEs remodeled their behavior to overlap and align; otherwise, the repelling pattern appeared between the distinct TEs.

This conclusion can be further validated in the scion or rootstock parts. For example, in group C grafts, the TEs were out of alignment, and the TEs above the interface could roll back to fuse with themselves, thus formed a circle TE (Figure 4K), further supporting that homogeneous TEs tended to form a connection. Furthermore, in the self-grafts of *At* or *Nb*, we also noticed that connection was much more well-performed in the homogeneous TEs (Supplementary Figures 4A–C), and morphologically different TEs tend not to seal completely (apparent space between them) (Supplementary Figure 4B). These results reinforced that homogenous TEs from both partners are the key to establishing compatible grafts.

### Spiraling a TE Bundle Was Associated With Grafting Failure

**Figure 5:**
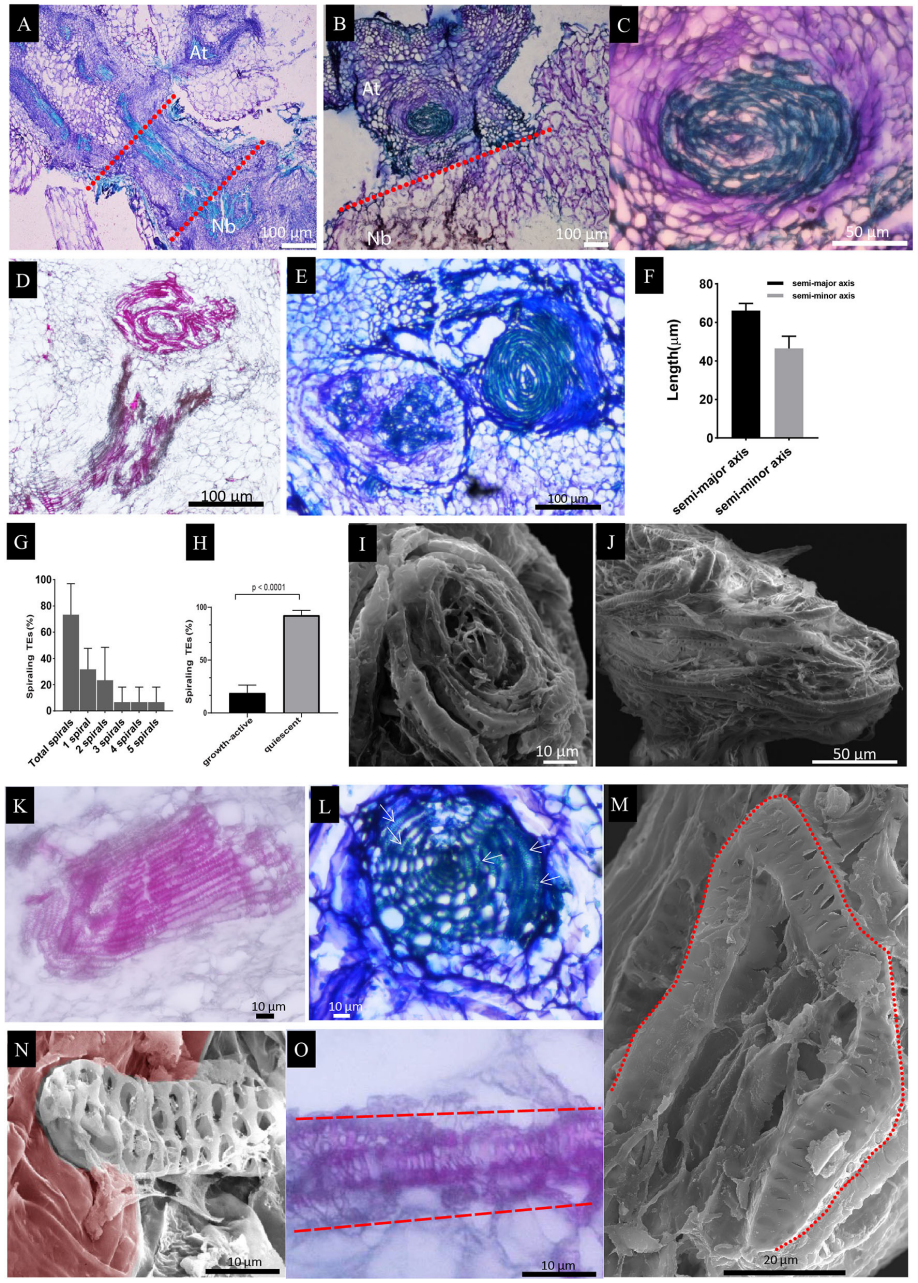
Histological staining of longitudinal sections in the *At*/*Nb* grafts and SEM observation of spiraling TEs. **(A)** A section showing the growth-active grafts (groups A and B). The dashed red line indicated the graft union. **(B)** A section showing the quiescent graft. **(C)** The enlarged spiraling structure in section B. **(D)** the spiraling structure of *At* and the xylem bundles of *Nb* (group C). **(E)** two spiraling structures were found above the graft union of quiescent graft (group C). **(F)** The average length of the semi-major and semi-minor axes in the spiraling structure. **(G)** Occurrence of various spirals in the examined grafts from 24 DAG to 30 DAG (three repeats, *n* = 11, 11, and 14, respectively). **(H)** The spiraling TEs in growth-active grafts and quiescent grafts at 30 DAG (*n* = 30 for growth-active grafts and *n* = 38 for quiescent grafts). **(I)** The vertically spiraling TEs in the tangential face of the xylem bundles from the quiescent grafts. **(J)** The horizontally spiraling TEs from the growth-active grafts. **(K)** The TE bundles of the rootstock protrude into the cortex tissue. **(L)** A web-like structure was found below the graft union of quiescent graft. The arrow indicated the rolled TEs. **(M)** Single-curled TE in the *Nb* rootstock. **(N)** A single TE protruding into the cortex tissue was observed under SEM. The cortex tissue was partly rendered brown red. **(O)** A single TE protruding into the cortex tissue was observed under a light microscope. Sections A, B, C, E, and L were stained by.5% toluidine blue O. Sections D, K, and O were stained by 1% Safranin O.

The scion and the rootstock from group C grafts were easily parted during the preparation for SEM, thus preventing further dissection of how the two parts have interacted. To resolve this issue, we performed longitudinal sectioning of both group A and group C grafts. Results showed that the TE bundles grew laterally instead of vertically, such that a cross-sectioning-like vasculature appeared (Figures 5A,B), consistent with the SEM photograph (Figures 4C,D). More interestingly, the lateral growth of TE bundles in the scion seemed confined to a local region above the graft union and eventually rolled into spiral-like bundles (Figures 5B–E), here named as “spiraling TEs.” The xylem bundles of rootstock traversed the graft union; however, these spiral-like bundles could not make a connection with them as shown in Figure 5D, suggesting the highly organized structure might be a closed system, thus rejecting connection with another xylem; this could be further evidenced by the existence of two independent spiral-like bundles, both stemming from the scion (Figure 5E), and the size of the spiral-like bundles (10997.04 ± 1330.38 μm^2^) that did not vary much (Figure 5F). These spiraling TEs were widespread, with more than 70% of *At*/*Nb* grafts over 24 DAG having such structures (Figure 5G), and we did not detect with histology such structures in any *At* or *Nb* self-grafts. To further exclude the possibility of their occurrence from the healing process, we did a series of SEM examination on the hypocotyl-cut plants without grafting and found the TEs at the cutting surface did not roll into the spiraling structure but extend to form the part of root systems (Supplementary Figure 5), suggesting the spiraling TEs could stem from scion-rootstock interaction. Indeed, nearly all quiescent grafts had them (Figure 5H), but the growth-active grafts from Groups A and B occurred with such structures at a much lower frequency (Figure 5H). Furthermore, SEM imaging showed that the spiraling TEs were placed longitudinally in the quiescent grafts (Figure 5I). Occasionally, the growth-active grafts also had such structures but either placed circumferentially (Figure 5J) or partially spiraled (Supplementary Figure 6A). Further dissection showed that the circumferentially spiraling TEs appeared not to interfere with the underneath TE fusion (Supplementary Figure 6B). Taken together, these findings indicated that the highly organized TE bundles were the result of *At*-*Nb* interaction and could lead to the quiescent state of group C grafts.

In the rootstock, some of the TE bundles also grew laterally and protruded into cortex tissue (Figure 5K). Most of them were circumferentially placed and organized into a web-like structure, which also contains spiraling TEs (Figure 5L). In some cases, a single TE was curled to form a spiraling structure (Figure 5M), or behaved in a way as the bundles protruded into the cortex, e.g., Figure 5N by SEM and Figure 5O by histological sectioning. The single TE was not detected in the scion, suggesting a behavioral difference of xylem elements between *At* and *Nb* during the formation of spiraling TEs.

### A GFP Signal Can Be Translocated Across the Graft Union Except That of the Quiescent Grafts

**Figure 6:**
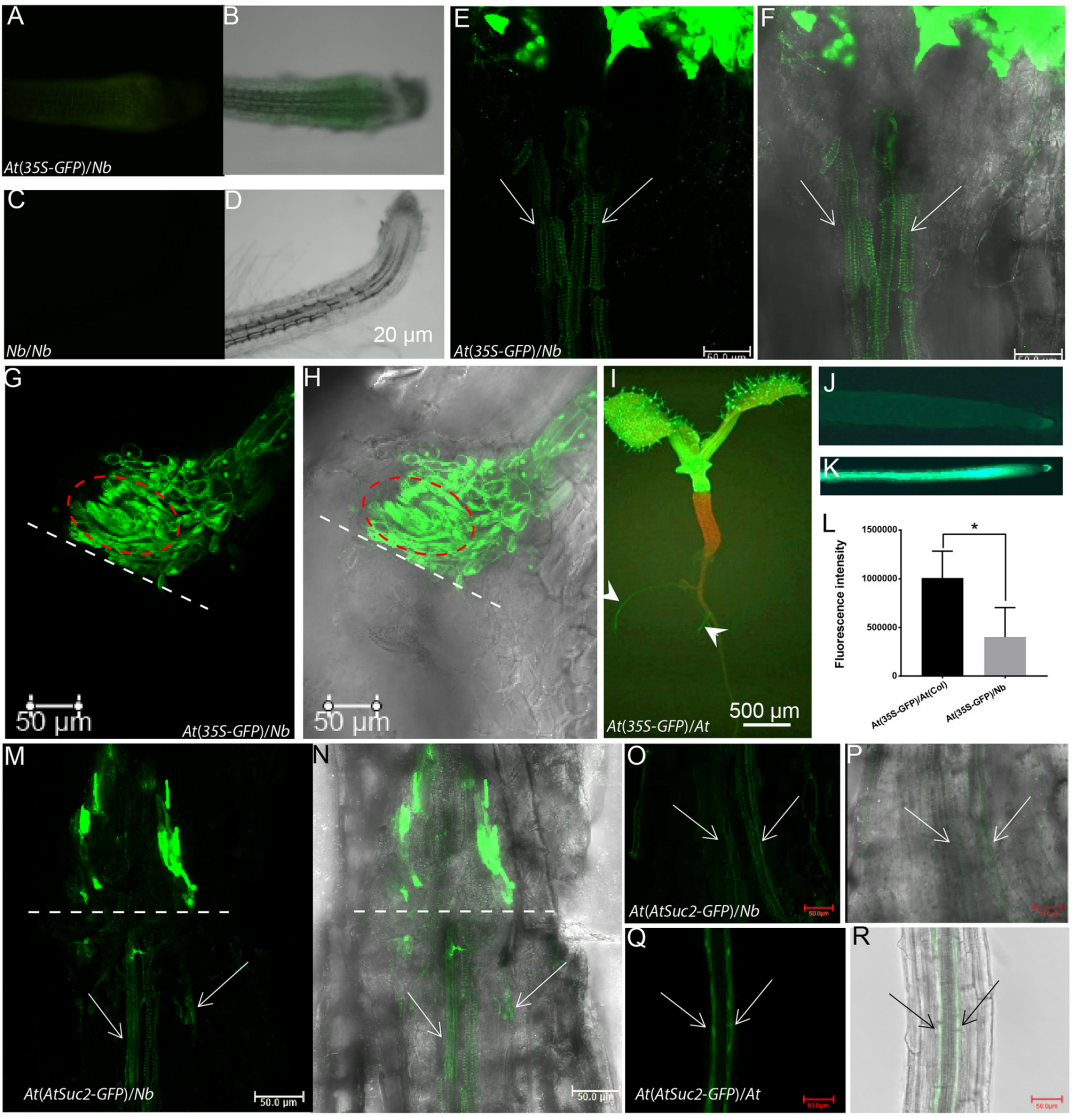
Translocation of GFP from *Arabidopsis* scion to *Nb* rootstock. **(A,B)** The root tip of an *At(35S-GFP)*/*Nb* graft at 15 DAG under fluorescence **(A)** and bright field (**B**, overlayed with **A**). **(C,D)** The root tip of *Nb* self-graft used as control. **(E,F)** GFP fluorescence was detected below the union of *At(35S-GFP)*/*Nb* growth-active grafts at 17 DAG with a Leica SP8 confocal microscope. The overlay image of a bright-field and green fluorescent (false color) channel was shown in **(F)**. The arrow indicated the GFP signal in the vascular bundles. **(G,H)** GFP fluorescence was restricted above the union of *At(35S-GFP)*/*Nb* quiescent grafts at 17 DAG. The bright-field and fluorescent overlay image was shown in **(H)**. The dotted line indicated the contacting interface. The circled area indicated the spiraling TEs. **(I)** An *At(35S-GFP)*/*At*(WT) self-graft at 7 DAG. The arrowhead indicated the GFP signal. **(J–L)** The GFP fluorescence comparison between *At(35S-GFP)*/*Nb* **(J)** and *At(35S-GFP)*/*At*(WT) graft **(K)**. **(L)** Comparison between fluorescence intensity of the rootstock of *At(35S-GFP)*/*Nb* and that of the *At(35S-GFP)*/*At*(WT) graft (*p* < 0.05). **(M,N)** The phloem-specific GFP fluorescence was detected below the union of *At*(*AtSuc2-GFP*)/*Nb* growth-active grafts at 25 DAG. **(N)** is the overlay image. **(O,P)** The GFP fluorescence was detected in the root phloem strands of growth-active *At*(*AtSuc2-GFP*)/*Nb* grafts. The arrows indicated the phloem strands of an *Nb* root. **(P)** is the overlay image. **(Q,R)** The GFP fluorescence was strongly detected in the root phloem strands of *At*(*AtSuc2-GFP*)/*At* self-graft. **(R)** is the overlay image. *p < 0.05.

To further explore the possibility of biomolecule movement in a growth-active *At*/*Nb* graft, we used *35S-GFP*-expressing *Arabidopsis* as a scion to test the shoot-to-root movement. At 15 DAG, a GFP fluorescent signal was detected in the root tip of *Nb* rootstock (Figures 6A,B) when compared with *Nb* self-graft control (Figures 6C,D). Furthermore, a longitudinal sectioning assay showed a GFP signal crossed the graft union and was presented in the vascular bundles of *Nb* rootstock hypocotyl at 17 DAG (Figures 6E,F). However, the GFP signal was rare if ever detected in the rootstock of quiescent grafts, and the blockage of downward movement of GFP was exactly correlated with the occurrence of spiraling TEs (Figures 6G,H), suggesting the bidirectional blockage *via* the formation of spiraling TEs.

In addition, the GFP signal was detected about 8 days earlier in the roots of *At*/*At* than in the roots of *At(35S-GFP)*/*Nb* (Figures 6A,B,I–K), and we also observed that the intensity of GFP fluorescence in the rootstock of *At(35S-GFP)*/*Nb* was lower than that of *At* self-grafts (Figures 6J–L). These results suggested that the phloem connection might be slower in the heterograft, given the scion-derived protein seemed mainly to move through phloem down to rootstock (Paultre et al., 2016). To confirm this result, we examined the movement of GFP driven by an *AtSUC2* promoter (Imlau et al., 1999). As seen in Figures 6M–P, the phloem-specific GFP signal could be detected in the hypocotyl and root phloem strands of *At(AtSuc2-GFP)*/*Nb* grafts but much weaker compared with the *At(AtSuc2-GFP)*/*At* self-grafts (Figures 6Q,R). These results indicated that scion-to-rootstock protein movement, indeed, occurred, although at a relatively slow pace, in the growth-active *At*/*Nb* heterograft.

## Discussion

As global interest in maintaining crop productivity under climate change intensifies, grafting as the traditional horticultural means provides an alternative approach to securing food security (Albacete et al., 2015). It has great potential to combine totally different genetic compositions with a biosymbiont to combat the complex threats posed by climate change and population growth. The wild and distant rootstocks that are more likely to confer a wide range of resistance to biotic and abiotic stressors could be used to impart beneficial traits to crop scion (Warschefsky et al., 2016). To reach this end, we must require a deeper understanding of the underlying processes of scion-rootstock interaction, primarily occurring at the graft union.

### TE Remodeling at the Grafting Union

Although the mechanisms to determine the grafting compatibility may vary between different species (Pina and Errea, 2005; Goldschmidt, 2014; Warschefsky et al., 2016), the vascular reconnection between scion and rootstock was considered as one of the critical steps toward establishing a compatible union (Pina and Errea, 2005; Melnyk, 2017; Wang et al., 2017). So far, only sporadic studies have examined the vascular behavior during the grafting process; for example, Copes (1975) showed that uneven xylem growth in graft union led to graft incompatibility in a pine tree. Subsequent work of Cope associated tracheid underproduction at the grafting interface with incompatibility (Copes, 1980). By analyzing the TE behavior in a time series after grafting, we found the small segmented TEs at the early stage of *At*/*Nb* union formation (within 7 DAG) may not be differentiated from a callus as commonly proposed in the literature (Moore, 1983; Pina and Errea, 2005; Baron et al., 2019). These shorter TEs apparently stemmed from longer single mature TE as evidenced in Figure 3A, showing that there were existing clear cracks between each individual segment. These segmented TEs were equipped with similar pits to the existing TEs (Figure 3A), suggesting the cell walls on these segmented TEs had undergone the wall-thickening process (secondary growth) to form the pits (Choat et al., 2008). Therefore, the small TEs within 7 DAG were unlikely to be differentiated from a callus in such a short-time window, given that the callus formation was much later and usually took 6–30 days (Ikeuchi et al., 2013), saving for the extra programming for vascular differentiation involving many steps, such as elongation, differentiation, and maturation with pits. We need to caution that short TEs at the later stage of graft union (more than 14 DAG) could be newly differentiated as the cell walls on these segments were smooth and possessed fewer pits (Figures 3C,D,G,H,K,L).

**Figure 7:**
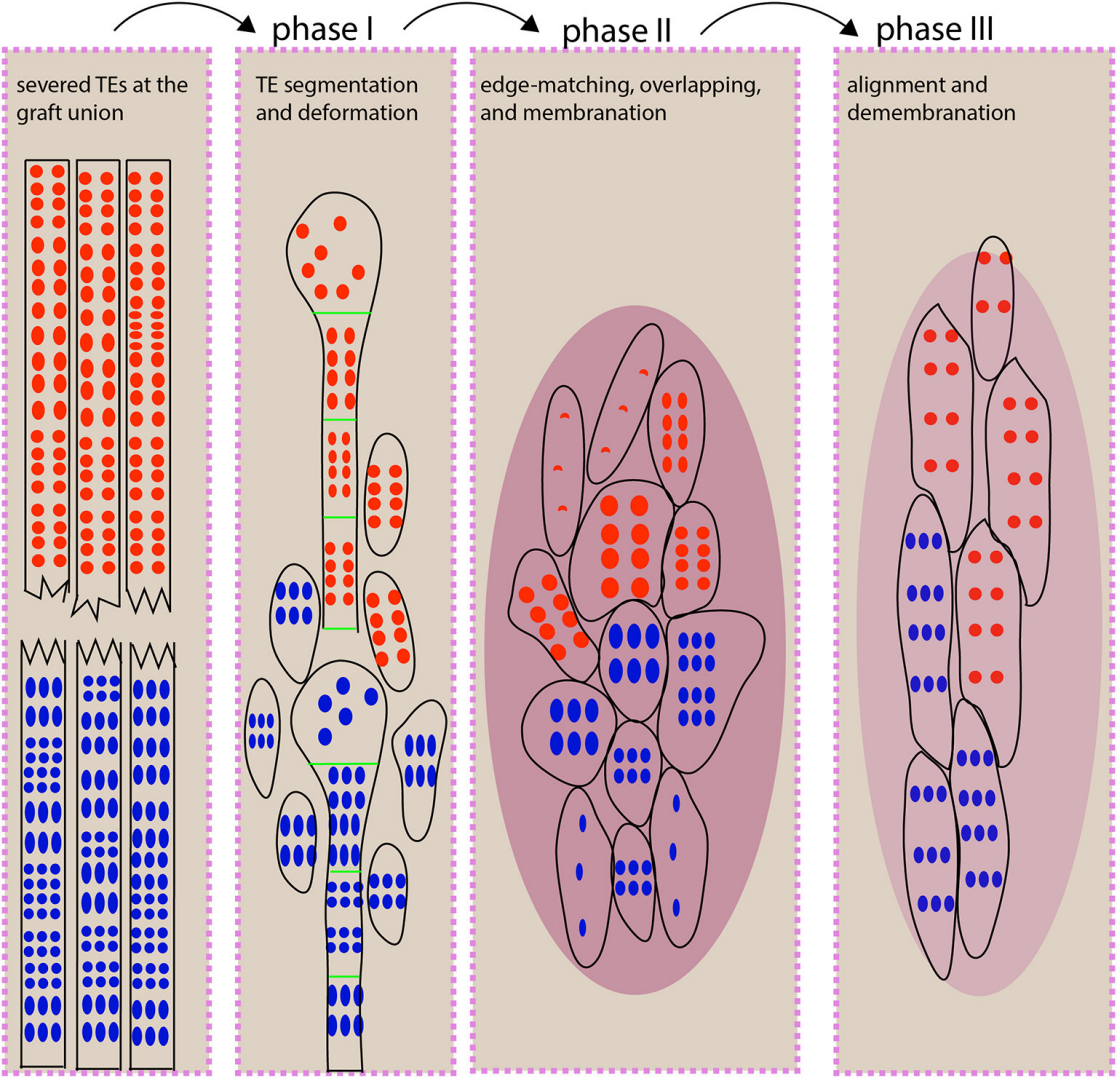
An illustration for TE behavior during scion-rootstock interaction. At the graft union, xylem connection depends on small, short TE behaviors, and this process can be distinguished into three phases. During phase I, the vascular strands at the grafting interface undergo segmentation and deformation (e.g., expansion of a TE diameter) to fill the gap between scion and rootstock. We propose that the segmented TEs may serve as timely “xylem fillers” that could provide a critical stopgap ahead of the full repair. In addition, the TE expansion leads to the increased surface area and volume, thereby increasing the likelihood of contacts. During phase II, the short TEs match with each other in a homogeneous way. The graft union is covered with membrane-like cellular deposits. Some newly differentiated TEs are beginning to form. During phase III, TEs are aligned vertically via fusion or overlapping to form a continuous network. The cellular deposits are withdrawn. The red and blue dots represent pits from scion and rootstock, respectively. The oval with few dots represents the newly differentiated TEs. The dark magenta oval represents cellular deposits. The light magenta oval represents reduced deposits.

The segmentation of long TEs into short TEs at the early stage of grafting (within 7 DAG) could significantly benefit the connecting process in two ways: (1) the increase in the width of TE (Figure 3) led to a squared increase of a surface and a cubed increase of TE volume, thereby enlarging the contact area and filling volume (Figure 7); (2) shorter vessels are more resistant to cavitation and an embolism than longer vessels (Cochard and Tyree, 1990; Lens et al., 2011; Christman et al., 2012; Jacobsen et al., 2018; Sergent et al., 2020), and prone to recover from an embolism occurring in stress conditions (Tyree and Yang, 1990). Because the segmented TEs were much smaller and their formation was also early (within 7 DAG) (Figure 3), we proposed that the segmented TEs could serve as “xylem fillers” that were employed to quickly fill the extra space between the scion and the rootstock (Figure 7), thus to effectively avoid escalating catastrophic xylem dysfunction arising from cavitation and an embolism introduced by grafting (Tyree and Sperry, 1989).

The connection between rootstock and scion requires the TEs overlapping in a homogeneously matching way. But the size and the pitting pattern of TEs are variable among and within species (Carlquist, 2001), and, indeed, we found the TEs of *At* mostly differ from that of *Nb* in size and shape (Figures 4F–H). These differences did not completely abrogate the TE connection between the two species. This is probably due to the similarity of the pitting type (in this case, the reticulate pitting (Figures 4A,B), which may weigh more important than other parameters such as a pit area and a TE diameter, etc. (Figures 4F,G). This could be comprehensible at the molecular level as various pitting patterns are, at least partially, determined by the organization of microtubules such as microtubule bundling (Pesquet et al., 2010), microtubule disassembly (Oda and Fukuda, 2012), and microtubule alignment (Sasaki et al., 2017), which, in turn, directs the deposition of both cellulosic and non-cellulosic materials on the cell wall, leaving the chemical composition of TEs non-homogenous. Thus, it would be very tempting to improve TE connection, thus grafting success by modifying the overall status of microtubules or changing the chemical deposition on the cell wall.

### A Structural Signature for Incompatible Grafts

One of the most advantages in studying the *At*/*Nb* grafting system lies in the observation that the compatible and incompatible grafts are both present, offering us a unique opportunity to compare the underlying processes that lead to the opposite grafting consequences. By comparison, we found that one of the key features in the incompatible grafts was the occurrence of the highly spiraled TE bundles (Figures 5B–E,I,J,L). These spiral-like structures interfered with vascular connection and made no connection with the TEs from the rootstock even though they were closely placed (Figures 4I, 5D), thereby strongly blocking symplastic and apoplastic movements (Figures 2E, 6G,H). In the compatible grafts, very rare spiraling TEs were detected; otherwise, only circumferentially spiraling TEs encircling the central pith were occasionally detected, indicating the peripheral occurrence of spiraling TEs may still allow the central xylem connection (Figure 5J), and the vertical spiraling TEs in the central pith led to unambiguous incompatibility (Figures 5B,D,E,I).

As for the origin of these spiraling bundles, they may most likely come from the fusion and circling of homogenous TEs based on the observation that the small circled TEs were formed from self-fused TEs due to the high homogeneity (Figure 4K). Then, the immediate question is why these TEs displayed an emergent behavior rather than a haphazard behavior. Exploring this question could involve looking at how TEs changed their growth state. Because of the intrusive growth, the major determinants of xylem and phloem fiber elongation in angiosperm species (Lev-Yadun, 2010; Snegireva et al., 2010; Gorshkova et al., 2012) were restrained within a certain space (Figures 5B–E); the transition from intrusive growth (Figures 5K,N) to spiraling growth is the key event to understanding the spiraling behavior and the underlying mechanisms of grafting incompatibility. With this clue, we suggest that future investigation would be laid on issues such as what factors arising from the process of xylem reconnection contributed to diverting TE elongation from intrusive growth to spiraling growth, thus to grafting incompatibility.

## Conclusion

In this study, we have used micrografting technique to present a heterografting system in which the grafted partners are genetically unrelated species, belonging to two taxonomic orders or families, respectively. A time-series comparison among quiescent grafts, growth-active grafts, and self-grafts revealed that a three-phase process allowed for TE connection between scion and rootstock (Figure 7), in which the homogenous TE overlapping was critical for a growth-active heterograft. Otherwise, the spiraling TE bundles developed in the quiescent grafts and blocked vascular connection. These distinct *At*/*Nb* grafting groups delivered deep insights into the TE behavior and cellular process at the grafting junction and might promise to provide continuing novelty on scion-rootstock interaction, particularly toward understanding the precise molecular process involved in grafting compatibility and incompatibility between taxonomically distant partners.

## Data Availability Statement

The original contributions presented in the study are included in the article/Supplementary Material, further inquiries can be directed to the corresponding author/s.

## Author Contributions

DL conceived the project and designed the experiments and wrote the manuscript. ZD, HW, TJ, TC, and MJ performed the experiments. ZD, HW, TJ, MW, and DL analyzed the data. All authors contributed to the article and approved the submitted version.

## Conflict of Interest

The authors declare that the research was conducted in the absence of any commercial or financial relationships that could be construed as a potential conflict of interest.
