# Supplementary material for: A Sequential Three-Phase Pathway Constitutes Tracheary Element Connection in the Arabidopsis/Nicotiana Interfamilial Grafts
Source: Front Plant Sci. 2021 Jul 5;12:664342. doi: 10.3389/fpls.2021.664342 (PMC8287886; doi:10.3389/fpls.2021.664342)
Supplement: Supplementary file 1 [file Data_Sheet_1.PDF]

Table S1. Adventitious roots of At/Nb grafts at 18 DAG

Number of grafts	adventitious root	Percentage %
105	10	9.52
67	12	17.91
327	90	27.52
186	54	29.03
211	61	28.91
