# Supplementary material for: A Sequential Three-Phase Pathway Constitutes Tracheary Element Connection in the Arabidopsis/Nicotiana Interfamilial Grafts
Source: Front Plant Sci. 2021 Jul 5;12:664342. doi: 10.3389/fpls.2021.664342 (PMC8287886; doi:10.3389/fpls.2021.664342)
Supplement: Supplementary file 2 [file Data_Sheet_2.PDF]

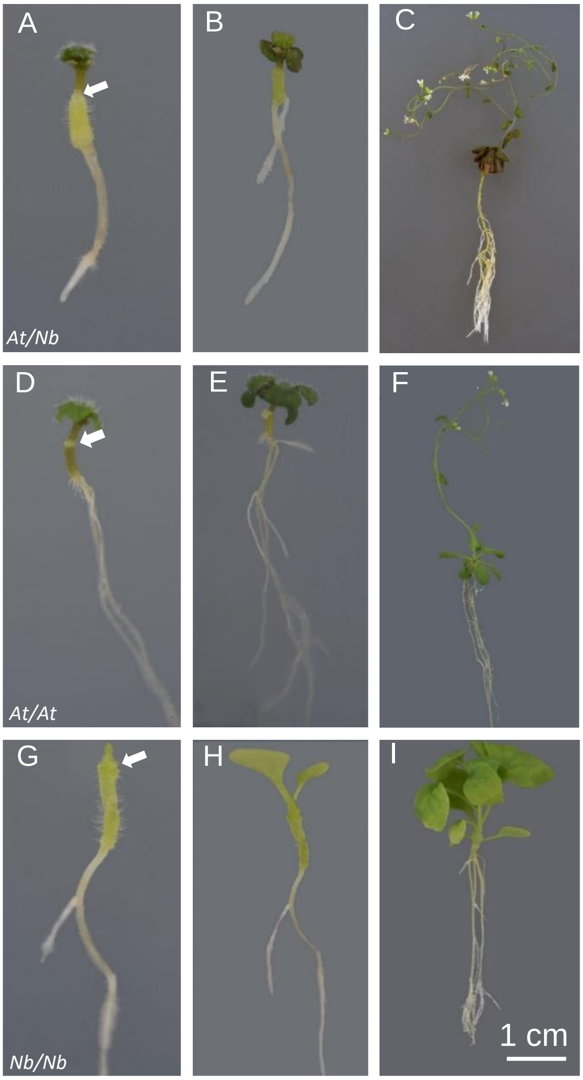

Figure S1. Phenotypic change of grafts in the different stage after grafting. The white arrow indicates the graft union. Phenotypic comparison between *Arabidopsis* self-grafts (D,E,F), *N. benthamiana* self-grafts (G,H,I), and *Arabidopsis* / *N. benthamiana* heterografts (A,B,C). (A,D,G), 7 DAG. (B,E,H), 15 DAG. (C,F,I) at flowering stage.

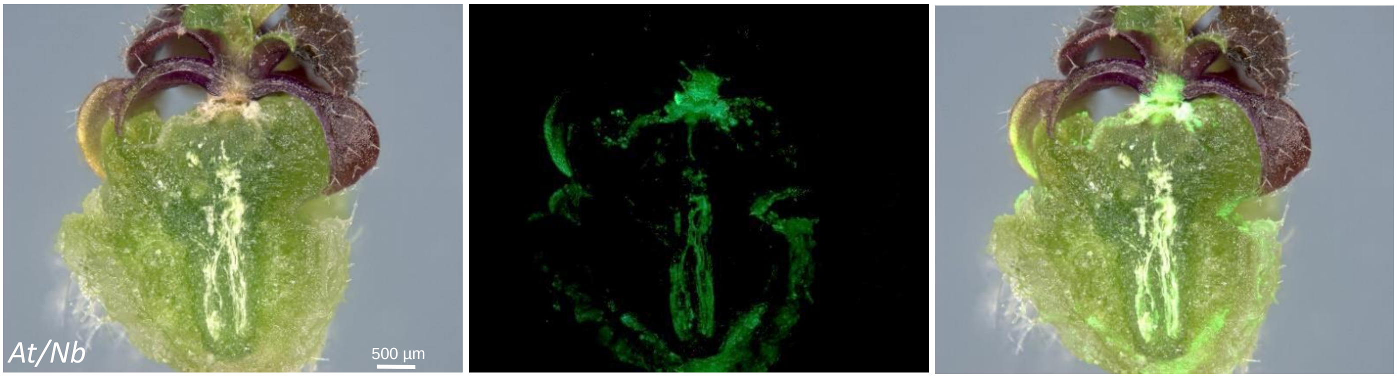

Figure S2. CFDA loading in the unsuccessful graft. The CFDA signal can be weakly detected in the phloem of some unsuccessful grafts.

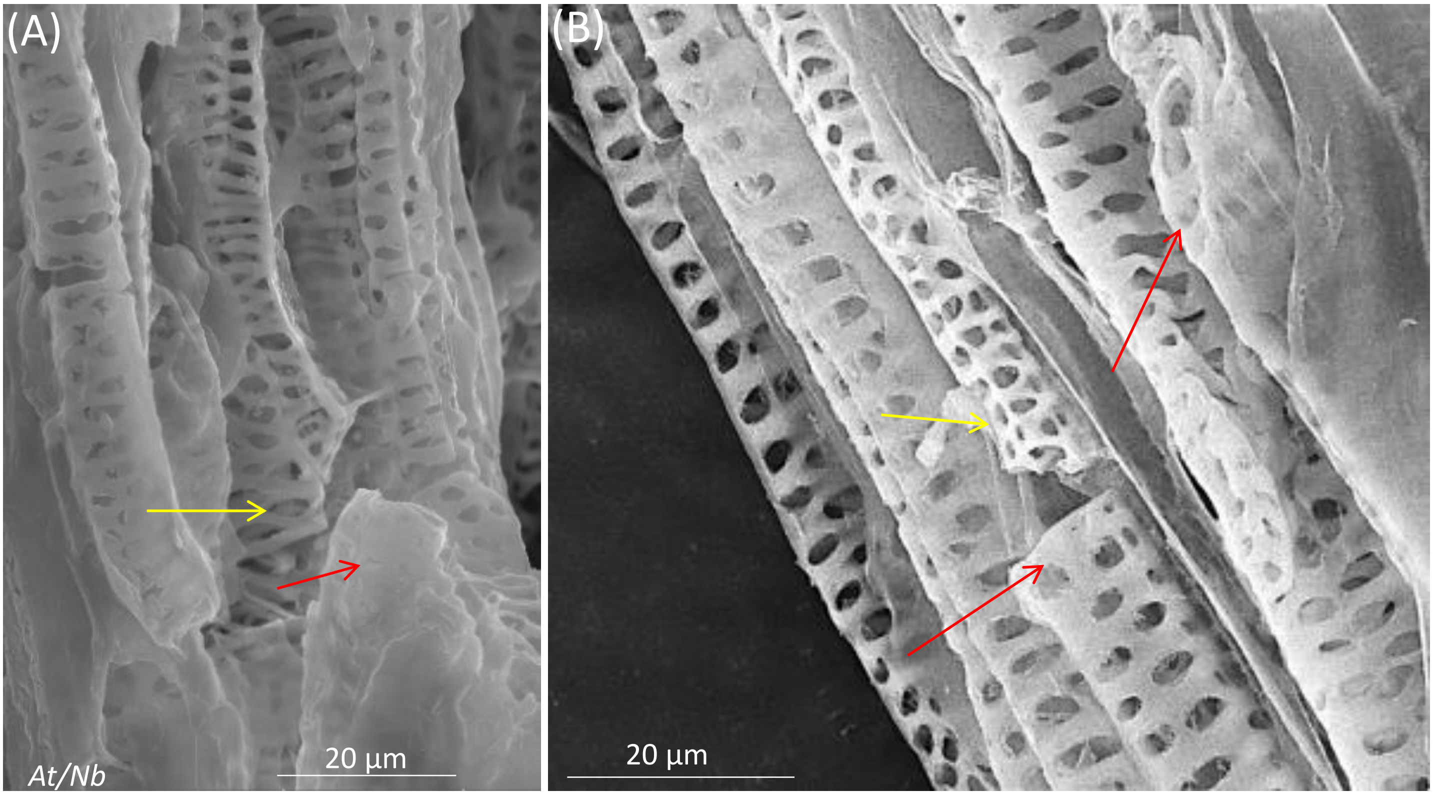

Figure S3. Non-homogenous tracheids at the grafting interface. (A) TEs with distinct pits including pit area and pit pattern did not connect to each other. (B) Two reticulate TEs with different diameter did not connect to each other. Yellow arrows indicate the TE of *At*. Red arrows indicate the TE of *Nb*.

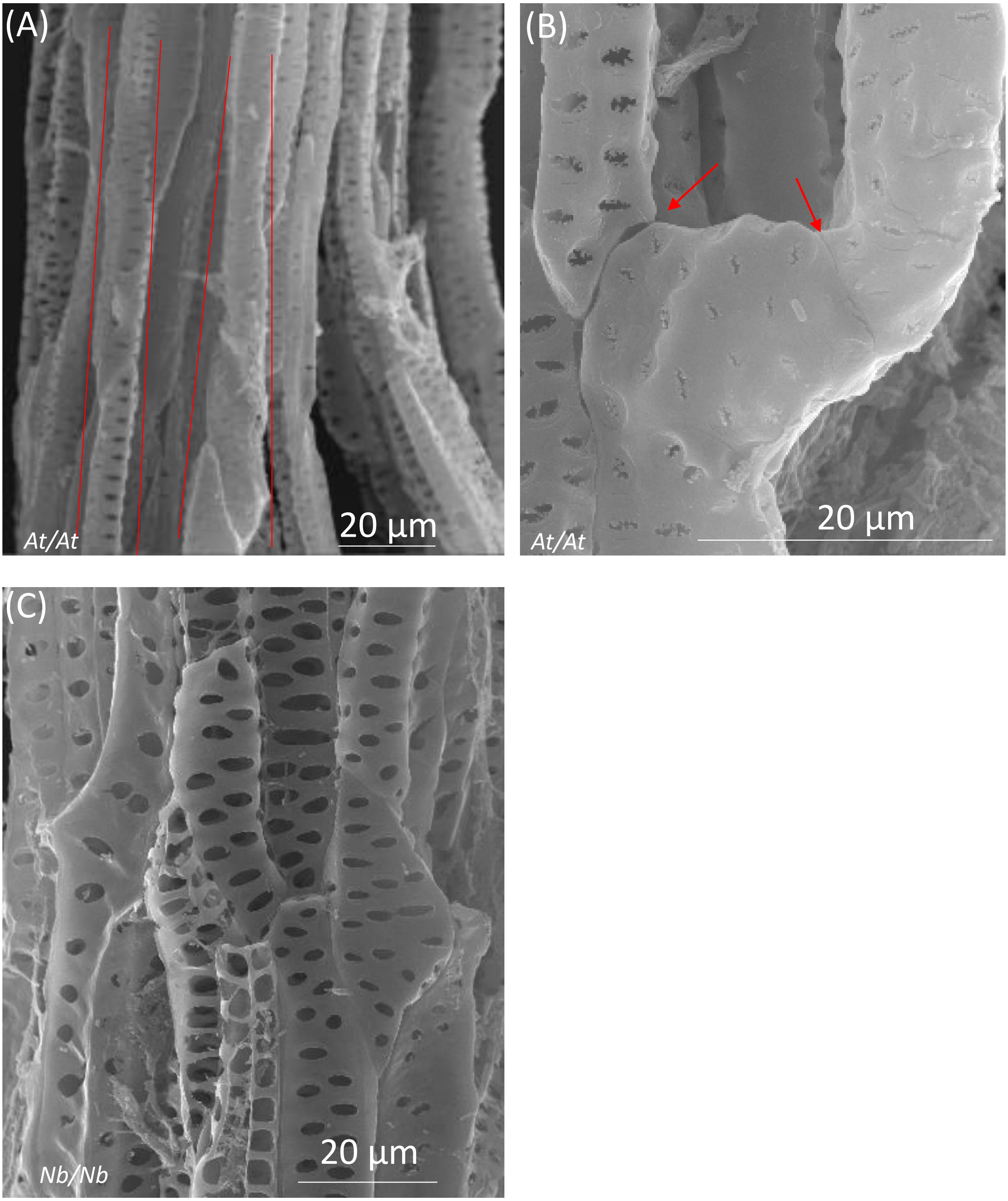

Figure S4. TE connection in *At* and *Nb* self-grafts. (A) In the *At* self-graft, the morphologically-similar tracheids were overlapping to form a continuous alignment. (B) Crack exists between non-homogeneous tracheids (left arrow) and a nearly seamless fusion (right arrow) between homogeneous tracheids in the *At* self-grafts. (C) The morphologically-similar tracheids were overlapping with each other, and also making head-to-head contacts in *Nb* self-grafts.

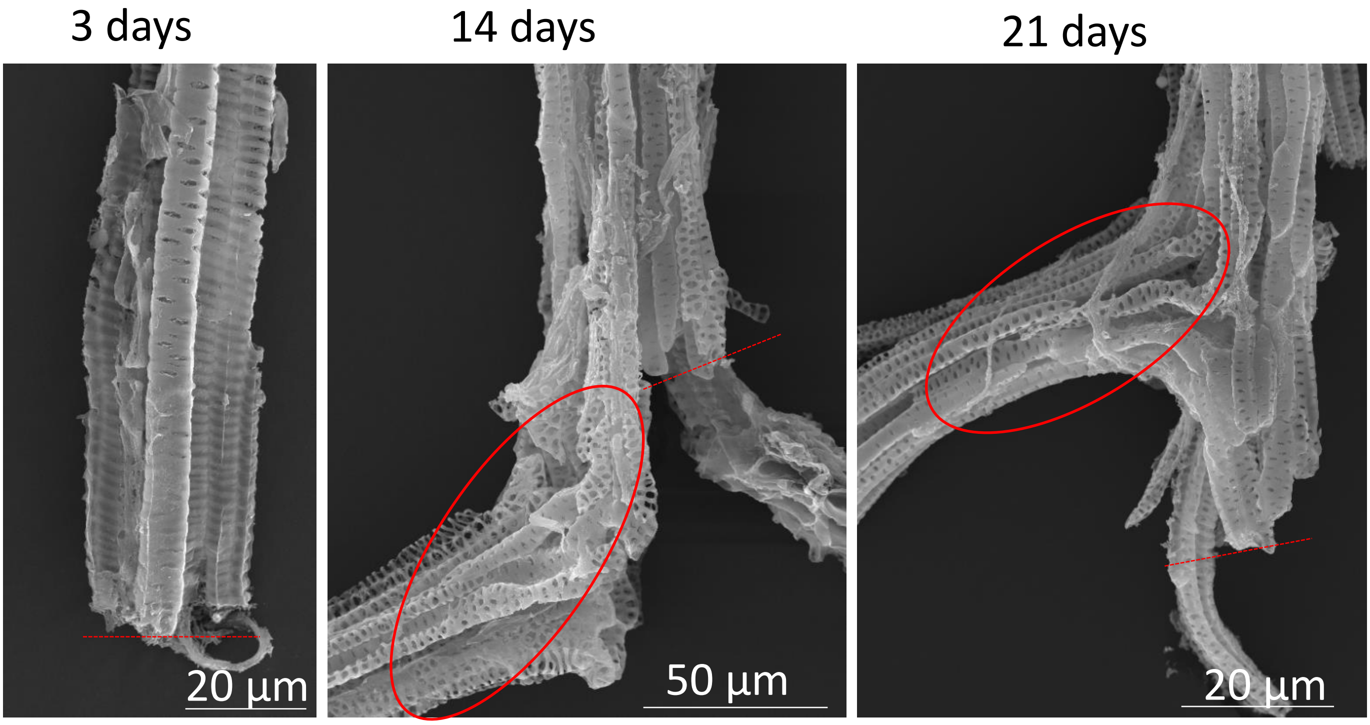

Figure S5. Xylem development after the lower part of *Arabidopsis* hypocotyl was removed. Without grafting, the newly developed vasculature emerged and no spiraling tracheid was detected. The oval indicates the newly formed xylem. The dashed line indicates the cut surface.

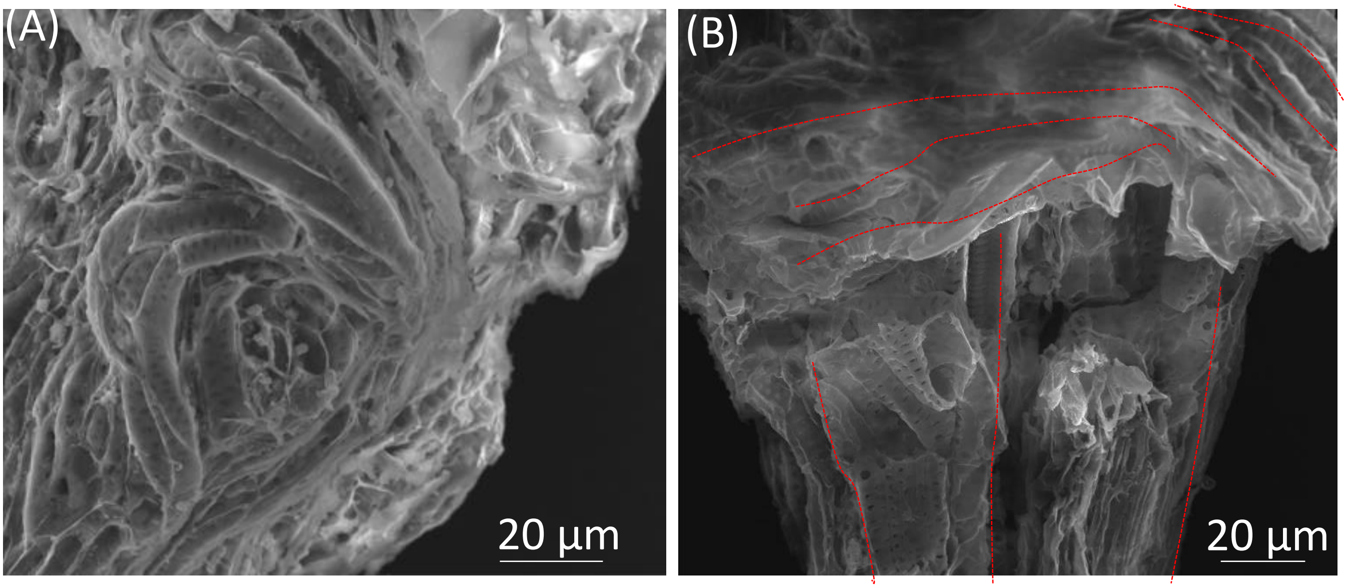

Figure S6. Spiraling structure in group A grafts. (A) A quasi-spiraling tracheids in the tangential face of the xylem bundles from the grafts in the group A grafts. (B) The inner connected tracheids under the horizontally spiraling tracheids.

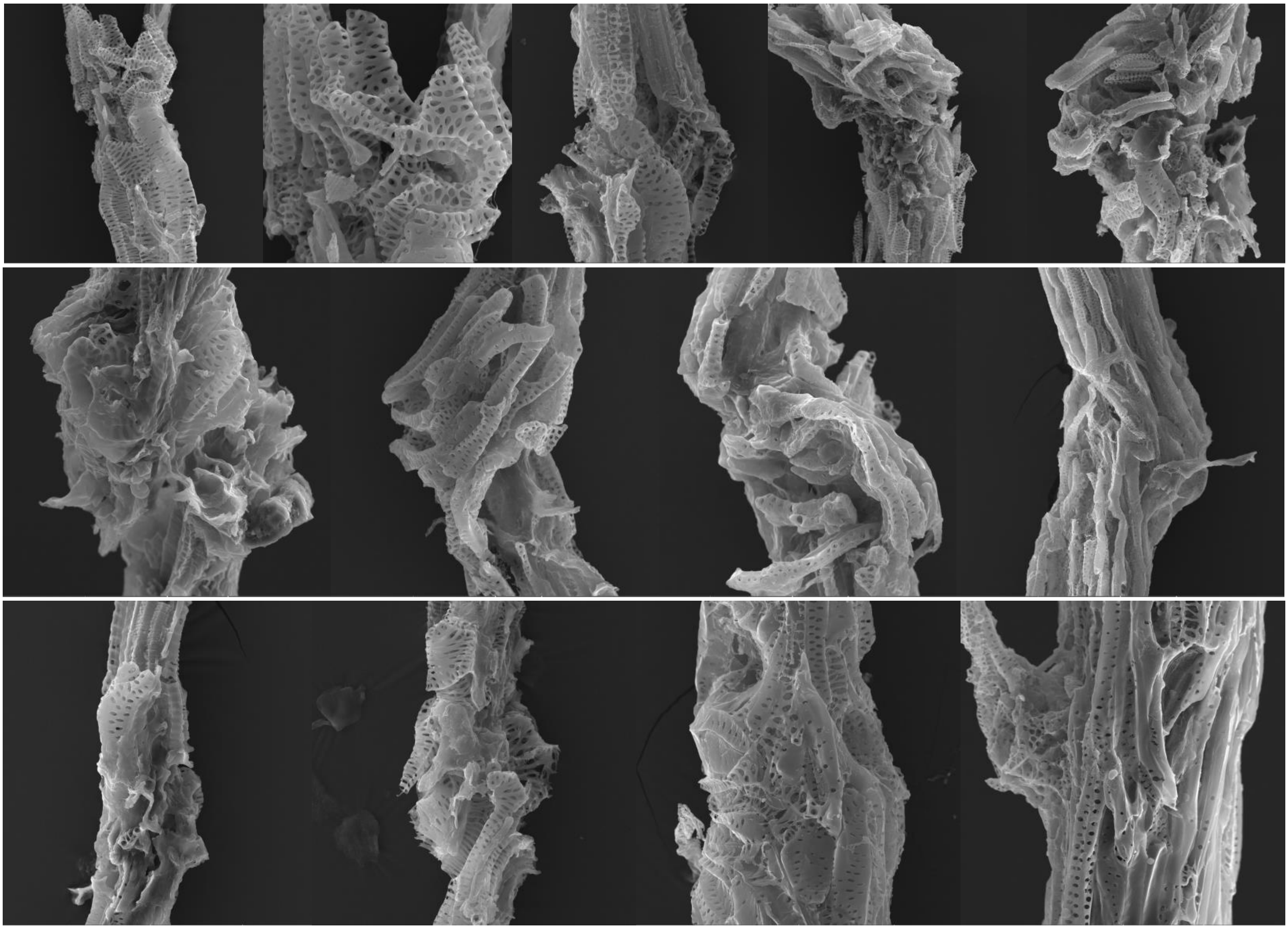

Figure S7. The original images to Fig. 3

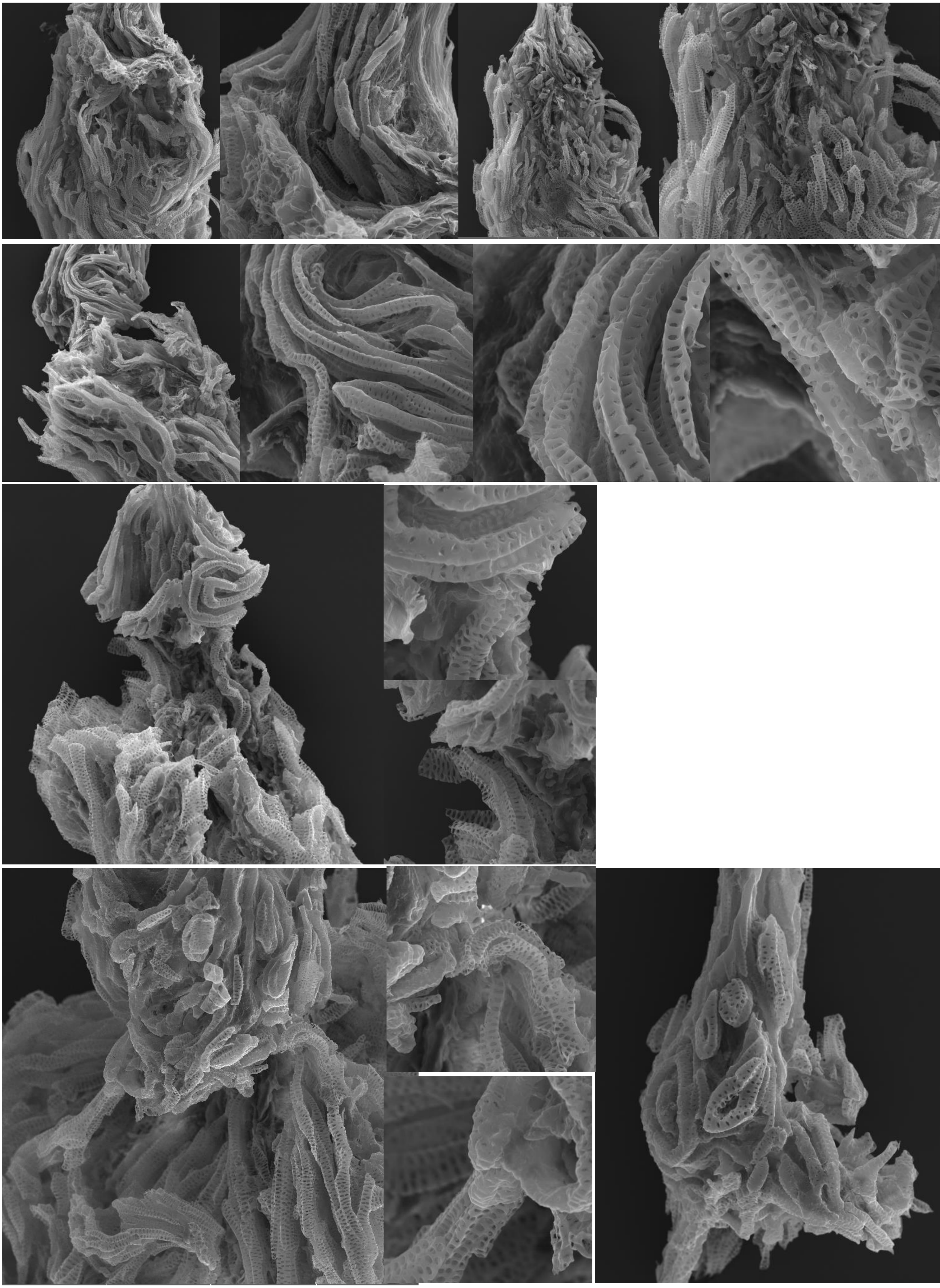

Figure S8. The original images to Fig. 4
